# Is Primary Open-Angle Glaucoma a Vascular Disease? Assessment of the Relationship between Retinal Arteriolar Morphology and Glaucoma Severity Using Adaptive Optics

**DOI:** 10.3390/jcm13020478

**Published:** 2024-01-15

**Authors:** Alina Szewczuk, Zbigniew M. Wawrzyniak, Jacek P. Szaflik, Anna Zaleska-Żmijewska

**Affiliations:** 1 Department of Ophthalmology, Public Ophthalmic Clinical Hospital (SPKSO), 00-576 Warsaw, Poland; 2 Faculty of Electronics and Information Technology, Warsaw University of Technology, 00-665 Warsaw, Poland; zbigniew.wawrzyniak@pw.edu.pl; 3 Department of Ophthalmology, Public Ophthalmic Clinical Hospital (SPKSO), Medical University of Warsaw, 02-091 Warsaw, Poland; jacek.szaflik@wum.edu.pl (J.P.S.); azaleska@wum.edu.pl (A.Z.-Ż.)

**Keywords:** ocular vascular disease, glaucoma, adaptive optics

## Abstract

Background: Retinal vascular abnormalities may be associated with glaucomatous damage. Adaptive optics (AO) is a new technology that enables the analysis of retinal vasculature at the cellular level in vivo. The purpose of this study was to evaluate retinal arteriolar parameters using the rtx1 adaptive optics fundus camera (AO-FC) in patients with primary open-angle glaucoma (POAG) at different stages and to investigate the relationship between these parameters and changes in spectral-domain optical coherence tomography (SD-OCT) and perimetry. Methods: Parameters of the retinal supratemporal and infratemporal arterioles (wall thickness (WT), lumen diameter (LD), total diameter (TD), wall-to-lumen ratio (WLR), and cross-sectional area of the vascular wall (WCSA)) were analysed with the rtx1 in 111 POAG eyes, which were divided into three groups according to the severity of the disease, and 70 healthy eyes. The associations between RTX1 values and the cup-to-disk ratio, SD-OCT parameters, and visual field parameters were assessed. Results: Compared with the control group, the POAG groups showed significantly smaller TD and LD values (*p* < 0.05) and significantly higher WLR and WT values (*p* < 0.05) for the supratemporal and infratemporal arterioles. TD was significantly positively correlated with the retinal nerve fibre layer (RNFL) and ganglion cell complex (GCC) (*p* < 0.05). LD was significantly positively correlated with the RNFL, GCC, and rim area (*p* < 0.05). The WLR was significantly negatively correlated with the RNFL, GCC, rim area, and MD (*p* < 0.05), while it was significantly positively correlated with the cup-to-disc ratio and PSD (*p* < 0.05). Conclusions: The results suggest that vascular dysfunction is present in POAG, even at a very early stage of glaucoma, and increases with the severity of the disease.

## 1. Introduction 

Glaucoma is the leading cause of irreversible blindness worldwide, but its aetiology is still not entirely determined [1]. Elevated intraocular pressure (IOP) remains the primary risk factor for glaucoma and the only modifiable risk factor that slows disease progression [1]. However, some patients experience disease progression despite significant IOP reductions [2,3]. Since the pathogenesis of glaucoma is multifactorial, other factors, such as reduced axoplasmic flow in retinal ganglion cell (RGC) axons, oxidative stress, and genetic background, may also play important roles [4,5]. Vascular abnormalities may also contribute to glaucomatous optic nerve damage [4,6]. The vascular theory postulates that reduced ocular perfusion flow (OPF) and impaired vascular autoregulation contribute to the progressive degeneration of RGCs through ischemic injury to the optic nerve [6]. Furthermore, other factors that affect OPF, including hypertension, diabetes, and migraines, are associated with the development of glaucoma, supporting the vascular theory [1,7,8]. The development of vascular hypotheses has accelerated over the past decade with new imaging techniques in ophthalmology. Changes leading to a reduced density and calibre of retinal blood vessels occur during glaucoma [1,6,7,8]. Numerous studies using optical coherence tomography angiography (OCTA) have shown significant reductions in the flow density, flow index, and vessel density in the optic nerve head and macula in patients with glaucoma compared to controls, as well as the association between these parameters and changes in the visual field [6,9,10,11,12]. Colour Doppler Imaging has demonstrated reduced velocities and an increased resistivity index in intraocular vessels in glaucoma patients [13]. Until recently, different techniques were used to analyse the calibre of retinal vessels [14]. Semi-automated software such as VAMPIRE (Vessel Assessment and Measurement Platform for Images of the Retina) [15] and Singapore I Vessel Assessment (SIVA) [16] have been developed to quantify retinal vascular parameters from digital retinal photographs. Also, studies have described the application of a retinal vessel analyser [17], spectral-domain optical coherence tomography (SD-OCT) [18], and scanning laser ophthalmoscopy (SLO) [19] for assessing the calibre of retinal vessels. However, only adaptive optics (AO) makes it possible to determine the thickness of the wall and the lumen diameter, as it can distinguish the vessel wall from the blood column [20]. AO has enabled noninvasive retinal examination at the cellular level with a resolution of about 2 μm by measuring wavefront distortions and compensating for them in real time with deformable mirrors [21,22]. AO alone does not produce an image and must be integrated with retinal imaging devices such as fundus cameras (FCs), SLO, and OCT. AO evaluates individual retinal structures, such as photoreceptors, blood vessels, nerve fibres, ganglion cells, the lamina cribrosa, and trabeculae in healthy eyes and various eye diseases [21]. The present study aimed to evaluate the parameters of retinal arteries in patients with POAG at different stages of progression using an rtx1 AO-FC, the first commercially available device integrated with AO. Secondly, we wanted to investigate the associations between these vascular parameters and glaucoma severity, defined by structural changes in OCT and functional changes in the perimetry. In addition, we wanted to compare the retinal vasculature of POAG patients and healthy subjects who were closely matched in terms of age and other parameters. This study is the first trial to analyse retinal vascular morphology using AO in correlation with other structural and functional tests in POAG.

## 2. Materials and Methods

The present study is a cross-sectional study with a single study visit, conducted between August 2021 and November 2022 at the Department of Ophthalmology, Faculty of Medicine, the Medical University of Warsaw, in the Ophthalmic Public Hospital in Warsaw. The Bioethical Commission of the Medical University of Warsaw approved the study protocol (approval number: KB/87/2015). All investigations were carried out according to the principles of the Helsinki Declaration. Written informed consent was obtained from all participants.

### 2.1. Subjects

We included 111 eyes of 58 POAG patients recruited from the Glaucoma Department at our hospital and 70 eyes of 38 healthy volunteers. Only one eye was included in the study for nine POAG subjects and six control subjects, as the other eye did not meet the inclusion criteria. All subjects were white Europeans over 18 years old. The patients included in this study had been diagnosed with bilateral POAG according to the requirements of the European Glaucoma Society Guidelines [23] with assessments of the RNFL, GCC defects, and ONH parameters via OCT and assessments of MD and PSD via perimetry. Glaucoma had been diagnosed and treated for at least two years. The glaucoma group was divided into three subgroups:

Group A—pre-perimetric glaucoma: no visual field scotoma in perimetry (37 eyes).

Groups B and C—perimetric glaucoma according to the criteria of a simplified Hodapp’s classification [23] divided as follows:

Group B—early glaucoma (48 eyes).

Group C—moderate glaucoma (26 eyes). 

In 24 patients, the glaucoma severity was at the same stage in both eyes; in the others, it was at different levels.

The control group was selected according to the following eligibility criteria: IOP < 21 mm Hg, normal appearance of the ONH, and normal OCT RNFL and GCC parameters. The inclusion parameters for all groups were as follows: absence of angle closure in gonioscopy, spherical lens less than six dioptres or cylindrical lens less than 2.5 dioptres, axial eye length less than 26 mm, best corrected visual acuity (BCVA) ≥ 0.4 on Snellen charts, explicit ocular media, high image quality, no history of intraocular surgery (excluding uncomplicated cataract surgery or uncomplicated glaucoma surgery for the glaucoma cohort), absence of diabetes mellitus, no history of trauma or other ocular diseases, and a lack of signs indicating secondary glaucoma for the POAG group.

### 2.2. Examination

All patients underwent an ophthalmic examination, including BCVA, refractometry, slit-lamp biomicroscopy, Goldmann applanation tonometry, gonioscopy, and direct fundoscopy. Axial eye length was acquired using an IOL Master 700 (Carl Zeiss Meditec AG, Hennigsdorf, Germany). The RNFL, GCC, thickness, and rim area were obtained using a spectral-domain OCT (SD-OCT) RTVue XR 100 Avanti Edition (Optovue, Fremont, CA, USA). A Humphrey 24.2 Sita standard visual field test with the reliability indices of the European Glaucoma Society was performed on a Humphrey Field Analyzer 3 (Zeiss, Oberkochen, Germany). All participants were asked to fill in a questionnaire about their characteristics, including age, sex, weight, height, and history of diseases, including hypertension, diabetes, hypercholesterolemia, arrhythmia, cardiovascular diseases (stroke and infarction), and smoking status. In addition, systolic blood pressure (SBP) and diastolic blood pressure (DBP) were measured in the sitting position on the brachial artery.

AO fundus images were obtained using an AO-FC (Rtx1™; Imagine Eyes, Orsay, France; version 3.4, also known as AO Image 3.4). Rtx1™ uses infrared light (850 nm wavelength) and is characterised by a resolution of 1.6 μm. The image dimensions are 4° × 4°, representing 1.2 mm × 1.2 mm of the retina. Image acquisition in a single position lasts approximately 4 s, during which 40 individual images are acquired [22,24]. The Rtx1™ software provides the program AO Detect Artery to analyse retinal vessel parameters and correct distortions within frames [22,25]. Most examinations were performed without pupil dilation; if high-quality image acquisition was not possible, pupil dilation was obtained with topical 1% tropicamide (Polfa, Warszawa). Images of the superior and inferior temporal retinal arterioles were obtained at 0.5–1 disc diameters from the edge of the optic nerve disc, that is to say, about 0.9–1.8 mm from the edge of the disc (an Rtx1 image is 1.2 mm × 1.2 mm), avoiding arteriovenous junctions and the adjacency of retinal veins. Furthermore, the occurrence of arteriovenous crossing and the pulsation of the retinal arteriole were infrequent in this region and consequently exerted minimal impacts on the measurements [26]. The following parameters were assessed to evaluate vessel morphology: lumen diameter (LD), wall thickness (WT), and total diameter (TD) calculated as single WT plus LD plus single WT (TD = WT + (WT + LD)). The wall-to-lumen ratio (WLR) and the cross-sectional area of the vascular wall (WCSA) were obtained automatically from the AO artery detection software version 3.4 (AO Image 3.4). The WLR is the ratio of the vessel’s WT to the LD, calculated as 2 × WT/LD, while the WCSA describes the relationship between the LD and TD. All the above-mentioned retinal parameters were measured three times on the scan with the best quality; the arithmetic mean of these three values was used in the statistical analysis. 

### 2.3. Statistical Analyses

The data analysis was conducted using Statistica^TM^ v. 13.2, TIBCO Software Inc., Palo Alto, CA, USA, 2017. Continuous variables, presented as means with their standard deviations (SDs), were compared between the POAG and control groups using either Student’s *t*-test or the Mann–Whitney U-test, depending on the data distribution. The Shapiro–Wilk test was used to determine the normality of each continuous variable. The Kruskal–Wallis and chi-square tests were used to compare at least three groups in terms of the quantitative variable. Relationships between numerical variables were assessed using a Pearson correlation analysis when the data met parametric test conditions and using a Spearman correlation analysis when they did not. A two-sided test was applied for *p*-values, and statistical significance was defined as *p* < 0.05. 

## 3. Results

### Comparison of General Data

The results for age, sex, BMI, SBP, and DBP showed no significant differences between all groups (*p* > 0.05). The mean intraocular pressure in all eyes was within normal limits, and the intraocular pressure was higher in the control group than in the glaucoma groups (*p* < 0.001 in the Kruskal–Wallis test). In terms of AL, there was a significant difference between group B and the control group (*p* < 0.001), but otherwise, there were no significant differences between the groups (*p* > 0.05). All groups were comparable to each other for the percentage of smokers, patients diagnosed with hypertension and hypercholesterolemia, and patients with a history of stroke or heart attack (*p* > 0.05). The clinical characteristics of the groups are presented in Table 1. 

The glaucoma groups were compared regarding the cup-to-disc ratio, estimated during direct fundoscopy; OCT parameters (mean RNFL, mean GCC, and rim area); and visual field parameters (mean deviation (MD) and pattern standard deviation (PSD)). The results are presented in Table 2. 

In addition, the course of glaucoma treatment is shown in Table 3. 

In the POAG groups (from A to C), there were significant decreases in the mean RNFL thickness (with a significant difference for groups A and B vs. C (*p* < 0.001)) and the mean GCC thickness (with a significant difference for groups A and B vs. C (*p* < 0.001)). We also observed rim area narrowing in the POAG groups from A to C, with a significant difference for groups A and B vs. C (*p* < 0.001 and *p* = 0.005, respectively), as well as a c/d ratio increase, with a significant difference for groups A and B vs. C (*p* < 0.001). The perimetry in the POAG groups showed an increase in PSD values, with significant differences between all groups (*p* < 0.001), and a decrease in MD values, with significant differences between all groups (*p* < 0.001). 

**Comparison of supratemporal retinal arterioles between POAG groups and the control group.**

The results are presented in Table 4.

**Figure 1:**
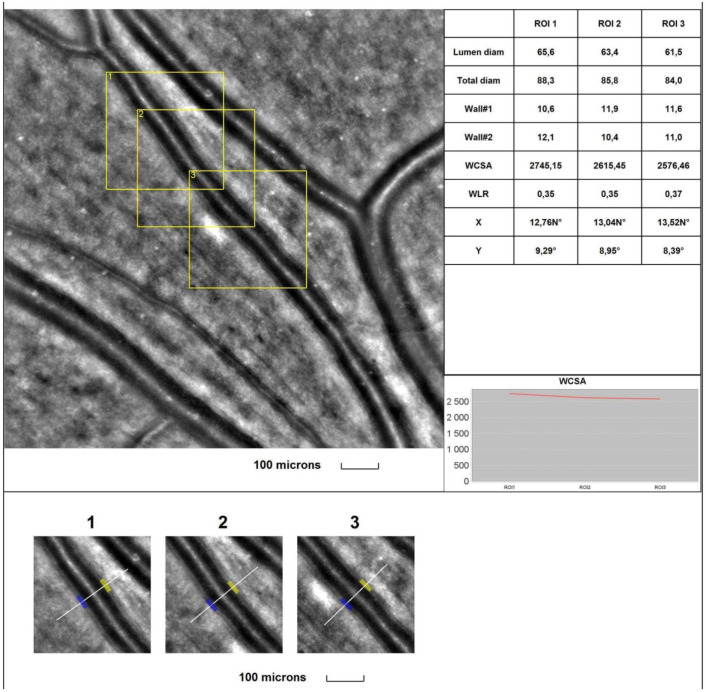
**Image of the supratemporal arteriole in a patient with primary open-angle glaucoma (POAG).** Evaluation of retinal arteriolar morphology in a POAG patient with adaptive optics camera using 4° × 4° square (Rtx-1, Imagine Eyes, Orsay, France) and measurement of morphological parameters using AOdetect software (AO Image 3.4). The parameters were calculated from the three selected regions of interest (yellow squares) for each time landmark (each with 100 μm width and height) (blue and yellow boxes indicate the walls of the arteriole) (**bottom**). The chart presents the following parameters: Lumen diam—lumen diameter; Total diam—total diameter; wall1 and wall2; WCSA—cross-sectional area; WLR—wall-to-lumen ratio. The image is from the author’s collection.

**Figure 2:**
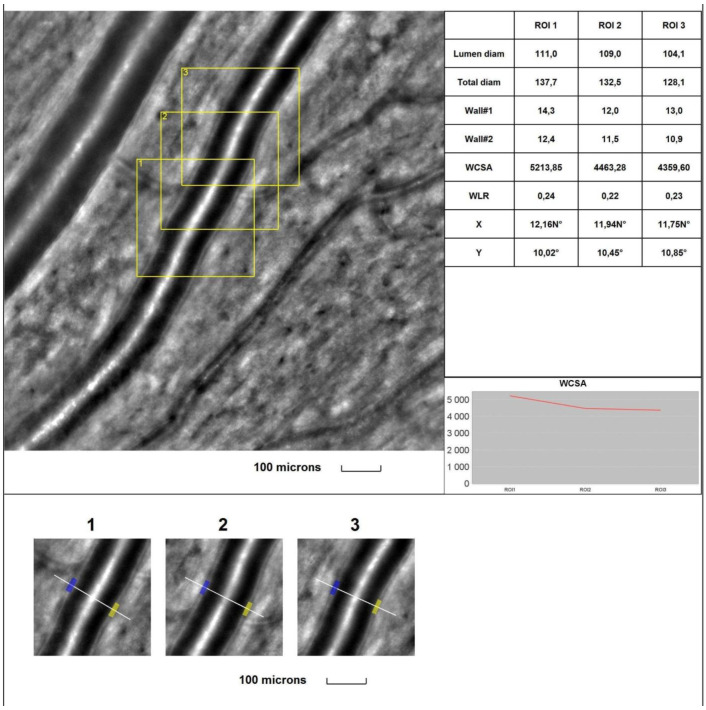
**Image of the supratemporal arteriole in a healthy subject.** Evaluation of retinal arteriolar morphology in a POAG patient with adaptive optics camera using 4° × 4° square (Rtx-1, Imagine Eyes, Orsay, France) and measurement of morphological parameters using AOdetect software (AO Image 3.4). The parameters were calculated from the three selected regions of interest (yellow squares) for each time landmark (each with 100 μm width and height) (blue and yellow boxes indicate the walls of the arteriole) (**bottom**). The chart presents the following parameters: Lumen diam—lumen diameter; Total diam—total diameter; wall1 and wall2; WCSA—cross-sectional area; WLR—wall-to-lumen ratio. The image is from the author’s collection.

The mean 1WT values **were significantly higher** in all glaucoma groups than in the control group (for A vs. control, *p* = 0.017; for B and C vs. control, *p* < 0.001). The mean 2WT values **were significantly higher** in all glaucoma groups than in the control group (for A, B, and C vs. control, *p* < 0.001). The mean WLR values **were significantly higher** in all glaucoma groups than in the control group (*p* < 0.001). The mean LD values **were significantly smaller** in all glaucoma groups than in the control group (*p* = 0.004, *p* = 0.011, and *p* < 0.001, respectively, for groups A, B, and C). The mean TD values **were significantly smaller** in all glaucoma groups than in the control group (*p* = 0.019, *p* = 0.044, and *p* = 0.003, respectively, for groups A, B, and C). **No statistically significant** differences were observed between the glaucoma and control groups for the mean WCSA (*p* = 0.604 in the Kruskal–Wallis test). Figure 1 and Figure 2 show the supratemporal arteriole parameters in a patient with POAG and a healthy subject, respectively. 

The results for the comparison of the infratemporal retinal arterioles between the POAG groups and the control group are presented in Table 4. 

**Figure 3:**
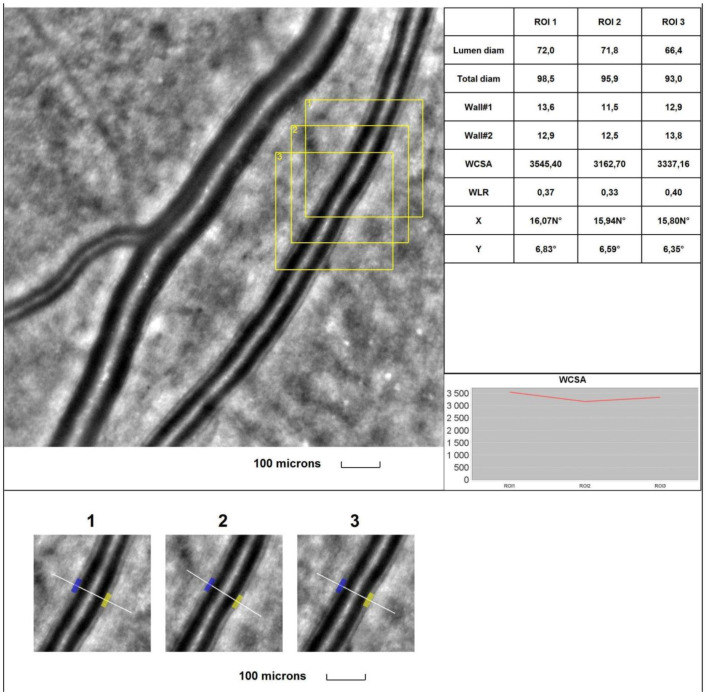
**Image of the infratemporal arteriole in a patient with primary open-angle glaucoma (POAG).** Evaluation of retinal arteriolar morphology in a POAG patient with adaptive optics camera using 4° × 4° square (Rtx-1, Imagine Eyes, Orsay, France) and measurement of morphological parameters using AOdetect software (AO Image 3.4). The parameters were calculated from the three selected regions of interest (yellow squares) for each time landmark (each with 100 μm width and height) (blue and yellow boxes indicate the walls of the arteriole) (**bottom**). The chart presents the following parameters: Lumen diam—lumen diameter; Total diam—total diameter; wall1 and wall2; WCSA—cross-sectional area; WLR—wall-to-lumen ratio. The image is from the author’s collection.

**Figure 4:**
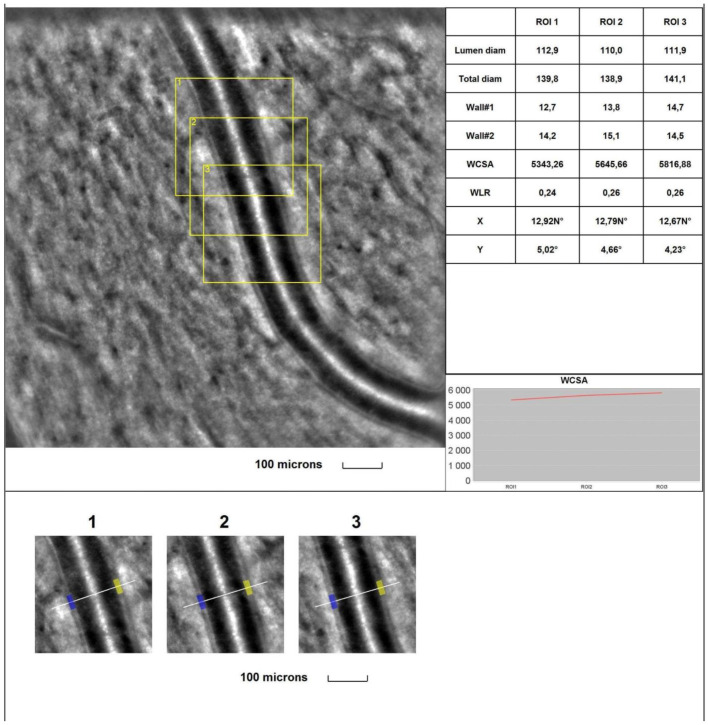
**Image of the infratemporal arteriole in a healthy subject.** Evaluation of retinal arteriolar morphology in a POAG patient with adaptive optics camera using 4° × 4° square (Rtx-1, Imagine Eyes, Orsay, France) and measurement of morphological parameters using AOdetect software (AO Image 3.4). The parameters were calculated from the three selected regions of interest (yellow squares) for each time landmark (each with 100 μm width and height) (blue and yellow boxes indicate the walls of the arteriole) (**bottom**). The chart presents the following parameters: Lumen diam—lumen diameter; Total diam—total diameter; wall1 and wall2; WCSA—cross-sectional area; WLR—wall-to-lumen ratio. The image is from the author’s collection.

The mean 1WT values **were significantly higher** in all glaucoma groups than in the control group (for A vs. control, *p* < 0.001; for B vs. control, *p* = 0.003; for C vs. control, *p* < 0.001). The mean 2WT values **were significantly higher** in all glaucoma groups vs. the control group (for A, B, and C vs. control, *p* < 0.001). The mean WLR values **were significantly higher** in all glaucoma groups than in the control group (for A vs. control, *p* < 0.001; for B vs. control, *p* < 0.001; for C vs. control, *p* < 0.001). The mean LD values **were significantly smaller** in all glaucoma groups than in the control group (for A, B, and C vs. control, *p* < 0.001). The mean TD values **were smaller** in all glaucoma groups than in the control group, but for group C, the difference was significant (for A vs. control, *p* = 0.087; for B vs. control, *p* = 0.054; for C vs. control, *p* = 0.008). **No statistically significant** differences were observed between the glaucoma groups and the control group for the mean WCSA (*p* = 0.248 in the Kruskal–Wallis test). Figure 3 and Figure 4 show the supratemporal arteriole parameters in a patient with POAG and a healthy subject, respectively. 

**Comparison of RTX 1 arteriolar parameters between glaucoma groups.** There were no significant differences in the rtx1 parameters between the glaucoma groups for the supratemporal (*p* > 0.05) and infratemporal (*p* > 0.05) retinal arterioles.

**Correlation analysis of supratemporal retinal arteriole parameters in POAG group.** TD was significantly positively correlated with the RNFL (r = 0.238) and rim area (r = 0.225, *p* < 0.05). LD was significantly positively correlated with the RNFL (r = 0.313), GCC (r = 0.199), and rim area (r = 0.265, *p* < 0.05). The WLR was significantly negatively correlated with the RNFL (r = −0.329), GCC (r = −0.265), rim area (r = −0.285), and MD (r = −0.290, *p* < 0.05), and it was significantly positively correlated with the c/d ratio (r = 0.191) and PSD (r = 0.353, *p* < 0.05).

**Correlation analysis of infratemporal retinal arteriole parameters in POAG group.** A weak positive correlation was found between the RNFL and LD (r = 0.193, *p* < 0.05). A significant negative correlation was noted between the mean RNFL and the WLR (r = −0.266, *p* < 0.05).

## 4. Discussion

Glaucoma is a multifactorial disease whose pathogenesis is not fully understood. Although the role of IOP is indisputable, other factors may also be involved, such as abnormal OBF and genetic factors [8]. It is suggested that altered OBF causes an unstable oxygen supply and thus may cause glaucoma damage [27,28]. Oxidative DNA damage can affect trabeculae, which induces resistance to aqueous humour outflow and RGCs, driving neurodegenerative changes [29,30]. Disruption of vascular autoregulation in ONH [27,31,32], higher retinal vascular resistance [33], and an imbalance between the vasoconstrictor ET-1 and the vasodilator nitric oxide have been observed in glaucoma [7]. Therefore, the evaluation of blood vessels in eyes with POAG is essential for a better understanding of the multifactorial pathogenesis of glaucoma. The high resolution of AO images makes them an ideal reference target for retinal vessel morphology measurements [34], with excellent intra-observer and inter-observer repeatability [35] and a good correlation with SD-OCT measurements [36].

The present AO study shows that retinal arterioles in glaucomatous patients differ from those in normal eyes. Each eye of the patients with glaucoma diagnoses was categorised into one of three groups according to the severity of the disease. Subgroup A included eyes without visual field defects with characteristic glaucomatous structural changes in ONH and SD-OCT. Subgroups B and C represented different stages of perimetric glaucoma. To enhance the statistical strength of the comparisons, we carefully matched all groups for age, BMI, and systemic blood pressure—well-known factors affecting retinal vascular calibres [20]. The average IOP in the adult population is estimated to be 15–16 mm Hg according to the EGS guidelines [23], and the results of our study are consistent with this statement. The lower IOP in glaucoma patients than in the control group may be explained by the therapy used to achieve the target IOP to slow the progression of the disease.

The total diameters (TDs) of the retinal arterioles were smaller in all glaucoma groups in both analysed locations: the supra- and infratemporal arterioles. Previously, the narrowing of retinal arterioles in POAG has been shown in numerous studies, mainly using colour fundus photographs [37,38,39,40,41]. In some studies, measurements have been taken using manual strategies [37,38,39], and in others, measurements have been taken with the help of semi-automatic software and standardised measurements [40,41]. In addition, the narrowing of retinal vessel diameters has been observed in glaucoma via SD-OCT using near-infrared images [42]. However, these methods did not provide information about the arteriolar lumen (LD). Our study showed significantly smaller LD values in all POAG groups compared to the control group. Our study confirms the result of Hugo et al., who were the first to use AO to evaluate the retinal vasculature in glaucoma and found a significant reduction in TD and LD in POAG patients compared to healthy individuals [14]. Our study also found that the WT and WLR values in the POAG group were significantly higher than in the control group. Hugo et al.’s previous study [14] does not support our results regarding the WT and WLR. Still, they analysed smaller groups (*n* = 31) than our study (*n* = 111), and there were no descriptions of structural changes in the OCT images or functional changes in the visual field.

On the other hand, the WLR and WT have been widely studied in other diseases, such as hypertension and diabetes. They showed significantly higher WT and WLR values in hypertensive [23,43] and diabetic [44] patients than in healthy subjects. By assessing both the WCSA and WLR, it is possible to distinguish between eutrophic and hypertrophic vascular remodelling [45]. Eutrophic remodelling is characterised by an increased WLR and an unchanged WCSA, as we obtained for the glaucoma patients in the present study. In eutrophic remodelling, the reduction in LD is caused by vasoconstriction by smooth muscle cells without a growth response [45]. On the contrary, hypertrophic remodelling is characterised by simultaneous increases in the WLR and WCSA caused by the growth of smooth muscle cells [45]. Therefore, the vascular morphological parameters in glaucoma (such as the WT, WLR, and WCSA) should be further investigated in larger groups and with consideration of other confounding factors.

Our study also showed significant positive correlations between TD and OCT parameters (RNFL and rim area) and between LD and OCT parameters (RNFL, GCC, and rim area) (*p* < 0.05). The WLR was significantly negatively correlated with the RNFL, GCC, rim area, and MD (*p* < 0.05) and was significantly positively correlated with the cup-to-disc ratio and PSD (*p* < 0.05). The WLR’s correlation with multiple determinants of glaucoma severity may make the WLR a good candidate biomarker for glaucoma progression. Our results support the concept that retinal vasoconstriction is associated with the progression of glaucoma measured via OCT and perimetry, as demonstrated in previous studies [41,46,47,48,49], and they are consistent with previous findings from cohort population-based studies, including the Blue Mountains Eye Study [50], the Beijing Eye Study [37], and the Singapore Malays Eye Study [51]. Furthermore, some prospective studies also support the concept of vascular alteration in glaucoma. Kawasaki et al. observed a prospective association between baseline arteriolar calibre narrowing and increased long-term risk of POAG in a cohort study over ten years [52]. Lin et al.’s study found that each standard deviation decrease in the baseline retinal vessel calibres was associated with a more than 30% increase in the risk of RNFL thinning and a more than 90% increase in the risk of VF deterioration during a 24-month follow-up period [53]. Yoo et al. investigated the retinal vessel diameter in patients classified as bilateral glaucoma suspects who showed unilateral glaucomatous conversion during a follow-up period of more than two years. There was a significant inter-eye difference in retinal arteriolar diameter at baseline between the eyes that converted to glaucoma and those that did not [54].

Further investigations of vascular changes in glaucoma would be helpful for better understanding its multifactorial pathogenesis, analysing the causes and effects of vascular and neurodegenerative changes in glaucoma, and the future invention of therapeutic targets, such as endothelin or Caveolin receptors, which can dilate retinal vessels and increase blood flow [2,55]. AO technology, due to its high resolution and image quality, allows the assessment of glaucomatous changes at the cellular level not only in the vessels but also in other structures such as nerve fibres [56,57,58], the lamina cribrosa [59], photoreceptors [60], trabeculae [61], and retinal ganglion cells [62,63]. In the future, AO may enable early glaucoma detection and more effective monitoring of the disease and treatment results. 

## 5. Limitations

This study has several limitations. First, the size of our study group could have been more significant. This study was conducted during the pandemic, which affected the size of the group. Second, patients with glaucoma under treatment were included, so the possibility that glaucoma medications may have affected the structure of the blood vessels cannot be ruled out. Third, we used both eyes of the subjects due to the different characteristics in each eye, but the same methodology was used for all groups (including those with healthy eyes). However, this might have affected the results of this study. This study was not prospective and cannot reflect long-term changes in blood vessels in patients with POAG.

## 6. Conclusions

The present study is the first assessment of microvascular morphology using AO in POAG in correlation with structural and functional parameters. Vascular changes in the early stages of the disease and their associations with disease progression are shown. This study may provide better insight into the pathogenesis and progression of POAG, which may contribute to better diagnosis and treatment of the disease.

## Figures and Tables

**Table 1:** Clinical characteristics of the groups.

Parameters	Group A	Group B	Group C	Control Group	*p*-Value ^†^
total number of eyes	37	48	26	70	0.370
age (m ± SD) (years)	51.1 ± 7.8	52.7 ± 8.2	53.5 ± 8.8	50.5 ± 5.9	0.232
sex (male/female)	12/25	24/24	11/15	26/44	0.489 0.559 ^§^
BMI (m ± SD) (kg/m^2^)	23.6 ± 2.4	24.3 ± 1.8	24.3 ± 2.0	23.8 ± 2.2	0.358
SBP (m ± SD) (cm Hg)	124.4 ± 11.4	126.7 ± 11.8	126.9 ± 9.9	121.5 ± 11.9	0.404 ^‡^
DBP (m ± SD) (cm Hg)	77.9 ± 8.4	78.4 ± 7.6	78.3 ± 7.3	78.7 ± 8.7	0.876
BCVA (m ± SD)	0.905 ± 0.118	0.908 ± 0.133	0.865 ± 0.147	0.989 ± 0.040	<0.001 ^†^ A **, B *, C *** vs. Control ***
IOP (m ± SD) (cm Hg)	13.2 ± 2.6	13.2 ± 2.5	13.0 ± 2.8	15.2 ± 2.5	<0.001 ^†^ A **, B **, C ** vs. Control **
AL (m ± SD) (mm)	24.0 ± 0.9	24.4 ± 1.2	23.9 ± 1.3	23.5 ± 0.9	0.905 ^‡^ B *** vs. Control ***
lens status (pseudophakic) (%)	2 (5.4%)	4 (8.3%)	5 (23.1%)	3 (4.3%)	

* *p* < 0.05; ** *p* < 0.01; *** *p* < 0.001; ^†^ Kruskal–Wallis test; ^‡^ chi-square test; ^§^ Mann–Whitney test for proportion of females and males. m—mean; SD—standard deviation; BMI—Body Mass Index; SBP—systolic blood pressure; DBP—diastolic blood pressure; BCVA—best corrected visual acuity; IOP—intraocular pressure; AL—axial length; *n*—number.

**Table 2:** Characteristics of the disease severity in the glaucoma groups.

Parameters	Group A	Group B	Group C	*p*-Value ^†^
Duration of glaucoma (m ± SD) (years)	7.1 ± 4.3	8.2 ± 5.2	9.0 ± 6.0	0.489
Cup-to-disc ratio (m ± SD)	0.554 ± 0.051	0.590 ± 0.031	0.815 ± 0.067	<0.001 A ***, B *** vs. C ***
RNFL (m ± SD) (µm)	87.3 ± 10.0	83.3 ± 10.7	72.6 ± 13.9	<0.001 A ***, B *** vs. C ***
GCC (m ± SD) (µm)	90.0 ± 11.2	84.4 ± 9.7	74.0 ± 13.7	<0.001 A ***, B *** vs. C ***
Rim area (m ± SD) (mm)	0.873 ± 0.262	0.857 ± 0.367	0.656 ± 0.381	<0.001 A ***, B ** vs. C ***
MD (m ± SD) (dB)	0.02 ± 1.02	−1.99 ± 1.74	−9.45 ± 2.95	<0.001 A *** vs. B ***, A *** vs. C ***, B ***vs. C ***
PSD (m ± SD) (dB)	1.58 ± 0.3	3.6 ± 1.9	10.54 ± 3.58	<0.001 A *** vs. B ***, A *** vs. C ***, B *** vs. C ***

** *p* < 0.01; *** *p* < 0.001; ^†^ Kruskal–Wallis test. m—mean; SD—standard deviation; RNFL—retinal nerve fibre layer; GCC—ganglion cell complex; MD—mean deviation; PSD—pattern standard deviation.

**Table 3:** Treatment history of glaucoma patients.

Parameters	Filtering Surgery (*n* (%))	Canaloplasty (*n* (%))	SLT (*n* (%))	Monotherapy (*n* (%))	Bitherapy (*n* (%))	Tritherapy (*n* (%))	Quadric Therapy (*n* (%))
Groups							
Group A	1 (2.7%)	4 (10.8%)	1 (2.7%)	21 (56.8%)	9 (24.3%)	1 (2.7%)	1 (2.7%)
Group B	6 (12.5%)	14 (29.2%)	2 (4.2%)	10 (20.8%)	13 (27.9%)	5 (10.4%)	0 (0.0%)
Group C	7 (18.2%)	6 (26.9%)	1 (3.8%)	5 (19.2%)	7 (26.9%)	5 (19.2%)	5 (7.7%)

*n*—number, SLT—selective laser trabeculoplasty. Anti-glaucoma medications included alpha two adrenergic agonists, beta-blockers, carbonic anhydrase inhibitors, and prostaglandins.

**Table 4:** Characteristics of rtx1 supratemporal and infratemporal retinal artery parameters.

Parameters	Group A	Group B	Group C	Control Group	*p*-Value ^†^
Supratemporal Arteriole					
1WT (m ± SD) (µm)	13.0 ± 2.0	13.1 ± 1.4	13.6 ± 2.0	12.0 ± 1.2	<0.001 A *, B ***, C *** vs. Control ***
2WT (m ± SD) (µm)	12.5 ± 1.3	12.9 ± 1.2	13.6 ± 1.7	11.4 ± 1.1	<0.001 A ***, B ***, C *** vs. Control ***
WLR (m ± SD)	0.294 ± 0.035	0.298 ± 0.044	0.329 ± 0.045	0.241 ± 0.023	<0.001 A ***, B ***, C *** vs. Control ***
LD (m ± SD) (µm)	87.8 ± 10.2	88.6 ± 11.1	79.9 ± 17.7	95.6 ± 15.2	<0.001 A **, B *, C *** vs. Control ***
TD (m ± SD) (µm)	112.3 ± 13.0	113.2 ± 12.3	110.5 ± 9.8	120.9 ± 11.9	0.001 A *, B *, C ** vs. Control **
WCSA (m ± SD) (µm^2^)	4041.9 ± 732.3	4161.3 ± 596.0	4148.1 ± 663.8	4033.5 ± 697.0	0.604
**Infratemporal arteriole**					
1WT (m ± SD) (µm)	13.6 ± 1.7	13.3 ± 2.1	14.1 ± 2.0	11.9 ± 1.3	<0.001 A ***, B **, C *** vs. Control ***
2WT (m ± SD) (µm)	13.7 ± 1.6	13.3 ± 1.7	13.81 ± 1.7	11.8 ± 1.2	<0.001 A ***, B ***, C *** vs. Control ***
WLR (m ± SD)	0.307 ± 0.034	0.298 ± 0.048	0.336 ± 0.047	0.239 ± 0.018	<0.001 A ***, B ***, C *** vs. Control ***, B *** vs. Control ***
LD (m ± SD) (µm)	90.3 ± 11.1	90.3 ± 9.7	84.2 ± 12.0	99.4 ± 10.3	<0.001 A ***, B ***, C *** vs. Control ***
TD (m ± SD) (µm)	116.6 ± 9.9	115.4 ± 13.2	111.2 ± 13.8	123.0 ± 11.6	0.003 C *** vs. Control ***
WCSA (m ± SD) (µm^2^)	4420.0 ± 712.7	4338.7 ± 801.9	4349.8 ± 913.6	4136.5 ± 688.6	0.248

* *p* < 0.05; ** *p* < 0.01; *** *p* < 0.001; ^†^ Kruskal–Wallis test. m—mean; SD—standard deviation; WT—wall thickness; WLR—wall-to-lumen ratio; LD—lumen diameter; TD—total diameter; WCSA—cross-sectional area of the vascular walls.

## Data Availability

The data presented in this study are available on request from the corresponding author.
